# RETRACTION: Evaluating the Effectiveness of Echocardiographic Guidance in Diminishing Postoperative Wound Complications for Left Atrial Appendage Closure: A Clinical Retrospective Study

**DOI:** 10.1111/iwj.70619

**Published:** 2025-04-21

## Abstract

**RETRACTION:** LiC.‐S. , LiX.‐F. , HuangX.‐Y. , and ChenF.‐F. , “Evaluating the Effectiveness of Echocardiographic Guidance in Diminishing Postoperative Wound Complications for Left Atrial Appendage Closure: A Clinical Retrospective Study,” International Wound Journal 21, no. 4 (2024): e14742, 10.1111/iwj.14742.38581265 PMC10998277

The above article, published online on 6 April 2024, in Wiley Online Library (http://onlinelibrary.wiley.com/), has been retracted by agreement between the journal Editor in Chief, Professor Keith Harding; and John Wiley & Sons Ltd. Following an investigation by the publisher, all parties have concluded that this article was accepted solely on the basis of a compromised peer review process. In addition, further investigation by the publisher found that the Materials and Methods provided incomplete details on the procedure and assessment of wound complications, that several references in the Discussion section did not support the statements being made, and the authors provided incomplete details on the ethical approval for the study. The editors have therefore decided to retract the article. The authors did not respond to our notice regarding the retraction.

**RETRACTION:** C.‐S. Li , X.‐F. Li , X.‐Y. Huang , and F.‐F. Chen , “Evaluating the Effectiveness of Echocardiographic Guidance in Diminishing Postoperative Wound Complications for Left Atrial Appendage Closure: A Clinical Retrospective Study,” International Wound Journal 21, no. 4 (2024): e14742, 10.1111/iwj.14742.38581265 PMC10998277

The above article, published online on 6 April 2024, in Wiley Online Library (http://onlinelibrary.wiley.com/), has been retracted by agreement between the journal Editor in Chief, Professor Keith Harding; and John Wiley & Sons Ltd. Following an investigation by the publisher, all parties have concluded that this article was accepted solely on the basis of a compromised peer review process. In addition, further investigation by the publisher found that the Materials and Methods provided incomplete details on the procedure and assessment of wound complications, that several references in the Discussion section did not support the statements being made, and the authors provided incomplete details on the ethical approval for the study. The editors have therefore decided to retract the article. The authors did not respond to our notice regarding the retraction.

